# Iron Stress Reprograms Enterocyte Metabolism

**DOI:** 10.3390/metabo15110691

**Published:** 2025-10-24

**Authors:** Shya E. Navazesh, Peng Ji

**Affiliations:** Department of Nutrition, University of California Davis, 1 Shields Ave., Davis, CA 95616, USA; senavazesh@ucdavis.edu

**Keywords:** iron deficiency, iron excess, enterocyte, IPEC-J2 cells, untargeted metabolomics, intestinal inflammation

## Abstract

**Objectives**: This study utilized IPEC-J2, a neonatal pig jejunum-derived cell line, to assess how iron deficiency (ID) and excess (IE) alter enterocyte metabolism and the transcription of inflammatory markers. **Methods**: Cells were treated with deferiprone (DFP) or ferric ammonium citrate (FAC) to induce ID or IE, respectively. The study evaluated: (1) transcriptional changes in iron-regulatory genes over 96 h under ID or IE; (2) the interaction between iron imbalance and lipopolysaccharide (LPS) exposure on mRNA expression of inflammation markers and iron transporters; and (3) cellular metabolic responses to ID, IE, and iron repletion using untargeted metabolomics. **Results**: ID triggered dynamic transcriptional changes in iron regulatory genes and suppressed cellular proliferation via impaired DNA replication. IE resulted in a persistent reduction in *TFRC* expression. LPS increased *CYBRD1* (*p* < 0.001) and *IL8* (*p* = 0.004) and tended to elevate *TLR4* and *TNF* expression (*p* ≤ 0.07), while iron deficiency upregulated *IL8* expression (*p* < 0.001). ID disrupted the TCA cycle, reduced glucuronic acid synthesis, and elevated glycolysis for energy production, whereas IE increased cholesterol biosynthesis and decreased alpha-tocopherol levels. Repletion of iron partially reversed ID-induced metabolic changes. **Conclusions**: ID impaired enterocyte proliferation and profoundly disrupted cellular metabolism, whereas IE enhanced cholesterol synthesis and depleted alpha-tocopherol levels. Restoration of cellular metabolism following iron repletion was observed, highlighting the resilience of enterocytes.

## 1. Introduction

Iron is an essential micronutrient required for cellular metabolism and various physiological processes. While indispensable throughout life, it is particularly critical during infancy to support rapid expansion of blood volume, growth, and neurodevelopment [1]. Chronic iron deficiency (ID) in young children has been linked to impaired cell-mediated immunity, increased infection risk, and stunted growth [2,3,4]. To mitigate these outcomes, iron fortification or supplementation strategies are often utilized during early infancy [5]. While these efforts may reduce the risk for iron deficiency, routine iron supplementation in non-anemic infants may lead to adverse effects such as reduced linear growth, diarrhea, and intestinal inflammation [6,7,8]. These developmental and gastrointestinal consequences are largely attributed to disrupted erythropoiesis and increased colonization of enteric pathogens; however, the impact of iron imbalance on intestinal epithelial cell function remains incompletely understood.

Enterocytes are responsible not only for nutrient absorption but also for mediating microbial-host crosstalk and maintaining intestinal barrier [9,10]. To ensure efficient absorption and barrier integrity, enterocytes are continuously renewed every 4–5 days. Beginning in the crypt as an absorptive progenitor cell, mature enterocytes migrate towards the tip of the villus, where they are eventually exfoliated due to apoptosis [11]. These short-lived cells exhibit high rates of glycolysis and heavily rely on glucose and glutamine to maintain their metabolic homeostasis and function in both humans and rodents [12,13].

Despite this continuous renewal, enterocytes are vulnerable to environmental insults; for example, exposure to enterotoxins downregulates nutrient transporter genes in Caco-2 cells and ileal enteroids, accompanied by disrupted brush border morphology and diminished expression of microvillus assembly genes [14]. Nutritional interventions, however, have been shown to improve metabolic function and enhance resilience [15,16,17,18]. Previously, supplementation of conditionally essential amino acids (glutamine or arginine) increased tight junction protein expression and promoted autophagic survival in stressed intestinal epithelial cell lines [13,15,16,17].

Among the nutrients that shape enterocyte function, iron plays a critical but tightly regulated role in cellular energy metabolism, redox balance, and immune signaling. Our previous research demonstrated that both cellular iron deficiency and overload reprogram intermediary metabolism and modulate immune responses in primary porcine alveolar macrophages (PAM), under both resting and LPS-stimulated states [19]. Iron-deficient PAMs accumulated TCA cycle intermediates and itaconate, an antimicrobial metabolite, while iron-overloaded PAMs exhibited increased cholesterol levels. Interestingly, both conditions dampened LPS-induced inflammatory responses [19].

In the intestinal epithelium, iron deficiency has been linked to villus atrophy, impaired nutrient absorption and increased barrier permeability [20,21]. In contrast, iron overload is known to promote reactive oxygen species (ROS) production, oxidative stress, lipid peroxidation, inflammation, and barrier dysfunction [20,21]. Iron-dependent lipid peroxidation is a central mediator of ferroptosis—a regulated non-apoptotic form of cell death increasingly recognized for its implication in intestinal diseases [22]. Despite these findings, a comprehensive characterization of how iron imbalance alters enterocyte metabolism during inflammation is still lacking.

Caco-2 cells are widely used to study nutritional modulation of gut epithelial functions. However, their colon carcinoma origin limits the physiological relevance to study the transcriptional and metabolic features of intestinal epithelial cells. Their anchorage-independent growth and resilience to attachment-regulated apoptosis differ markedly from primary enterocytes [23]. In contrast, porcine intestinal epithelial cell lines (IPEC-1 and IPEC-J2), especially the latter, derived from the neonatal pig jejunum, are non-transformed and non-tumorigenic, making them more suitable for modeling primary enterocytes [24]. Compared to the IPEC-1, IPEC-J2 cells exhibit higher oxidative phosphorylation and glycolytic activity, indicative of a more metabolically active phenotype [25]. These features make IPEC-J2 a valuable in vitro model for studying intestinal epithelial metabolism and stress response. Therefore, this study utilized IPEC-J2 cells to characterize intestinal epithelial iron metabolism and to investigate the metabolic and stress responses of intestinal epithelial cells to iron imbalance with or without endotoxin exposure.

## 2. Materials and Methods

### 2.1. Experimental Design

The study consisted of three experiments designed to evaluate (1) transcriptional changes in iron regulatory genes over time in response to iron over-exposure or deficiency, (2) the role of iron availability in regulating inflammatory responses to LPS treatment, and (3) metabolic adaption to iron deficiency and overload in IPEC-J2 cells (ACC 701; Leibniz Institute DSMZ, Braunschweig, Germany) in vitro. In all experiments, IPEC-J2 cells were cultured in Dulbecco’s Modified Eagle Medium/Nutrient Mixture F-12 supplemented with 10% Fetal Bovine Serum (Gibco, Waltham, MA, USA) and 1% Penicillin/Streptomycin (50 U/mL). Medium was changed every 2 days, and cells were passaged by trypsinization with 0.25% trypsin-EDTA (ThermoFisher Scientific, Rockford, IL, USA) upon reaching 70–80% confluence. Viable cell concentration was determined through 0.4% (*w*/*v*) trypan blue exclusion test using a hemocytometer. Cells were cultured in 6-well plates and experimental treatments were applied at approximately 80% confluence unless otherwise specified.

Ferric ammonium citrate (FAC) and iron chelator, deferiprone (DFP, ApexBio Technology, Boston, MA, USA), were used to induce cellular iron overload and deficiency, respectively. To identify effective doses without significant cytotoxicity (>80% viability), the viability of IPEC-J2 cells cultured under various concentrations of FAC (1, 10, 100, 1000 µM) or DFP (1, 10, 100, 1000 µM) was determined using XTT assay at 72 h of treatment. In addition, the protein expression of ferritin heavy chain (FTH) was determined using Western blot and used as a proxy to assess intracellular iron accumulation at 48 h of treatment. Using the established doses for FAC and DFP, we conducted the following experiments.

The first experiment aimed to characterize the temporal changes of iron transporter gene expression in response to iron overexposure and deficiency. IPEC-J2 cells were treated with complete culture medium as control (CON), or complete culture medium supplemented with 10 µM FAC or 500 µM DFP for 24, 48, 72, and 96 h. Total RNA was extracted from cells and reverse transcribed to cDNA for gene expression analysis using RT-qPCR. In the second experiment, we examined the interaction between iron status and inflammation on cellular proliferation rates and the expression of genes associated with iron metabolism and inflammatory responses. Cellular proliferation was measured using a bromodeoxyuridine (BrdU) assay (EMD Millipore Corp, Burlingame, MA, USA). Treatments included CON, 10 µM FAC, or 500 µM DFP for 48 h prior to being treated with 1 µg/mL lipopolysaccharide (LPS from *E. coli* O111: B4, Sigma-Aldrich, St. Louis, MO, USA) for 4 h. The final experiment investigated the cellular metabolome in response to iron excess, deficiency, and restoration by treating cells for 48 h with complete medium (iron-replete control, C), 10 µM FAC (iron excess, F), 500 µM DFP (deficiency, D), or exposing them to 500 µM DFP for 24 h followed by 10 µM FAC for 24 h (iron restoration from deficiency, DF). Cell lysates were analyzed for untargeted metabolomics at the West Coast Metabolomics Center at UC Davis. All experiments and assays were repeated or analyzed for 4 batches of cell cultures from different passages unless otherwise specified.

### 2.2. XTT Assay

An XTT cell viability assay (Biotium, Fremont, CA, USA) was used to determine the cytotoxicity of FAC and DFP at various concentrations. In this assay, dehydrogenase enzymes in metabolically active cells reduce the yellow tetrazolium salt, XTT, producing an orange formazan dye that is directly proportional to the number of viable cells. Cells in this experiment were cultured with XTT for 4 h at 37 °C in 96-well plates. Following incubation, absorbance was determined at 450 nm and corrected for background noise at 630 nm.

### 2.3. Total Protein Extraction and Western Blot

Protein extraction, electrophoresis, and Western blot procedures were performed as previously described [26]. Briefly, cells were rinsed in cold PBS and treated with RIPA lysis buffer (GenDepot, Katy, TX, USA) containing a cocktail of protease inhibitors (Thermo Scientific, Waltham, MA, USA). Cell lysates from three replicate wells were pooled together to harvest enough protein for analysis. Total protein was harvest after centrifugation and the concentration was determined using a Micro bicinchonic acid (BCA) protein assay (Thermo Scientific, Waltham, MA, USA). Samples were incubated at 60 °C for 60 min and then read at 562 nm.

Protein samples (15 µg) were separated on 10% APS stain-free gels (Bio-Rad Laboratories, Hercules, CA, USA) using SDS-PAGE (160 V for 55 min) in Tris-glycine running buffer containing 1% SDS. Proteins were transferred to nitrocellulose membranes using the Trans-Blot Turbo semidry transfer system (Bio-Rad, Hercules, CA, USA). Gel was activated under UV light to enable stain-free detection of total protein and imaged using a ChemiDoc MP system (Bio-Rad Laboratories) both before and after transfer to assess transfer efficiency. Membranes were blocked in TBST buffer containing 3% BSA for 1 h, then incubated overnight at 4 °C with rabbit polyclonal ferritin heavy chain antibody (1:1000, ab231253; Abcam, Cambridge, MA, USA) or rabbit polyclonal ferroportin antibody (1:1000, PA5-22993; Invitrogen, Waltham, MA, USA). After washes with PBST (PBS containing 0.1% Tween-20), membranes were incubated for 1 h at room temperature with HRP-conjugated goat anti-rabbit IgG (H + L) (1:20,000, 111-585-003; Jackson ImmunoResearch, West Grove, PA, USA) in blocking buffer, washed again, and developed with Clarity Western ECL substrate (Bio-Rad). The membranes were imaged using ChemiDoc MP (Bio-Rad, Hercules, CA, USA). Protein expression was normalized to total protein and quantified using ImageLab software (v.6.0; Bio-Rad).

### 2.4. RT-qPCR

At the end of the treatment, cells were immediately lysed and preserved in TRIzol reagent (Invitrogen, Waltham, MA, USA) and extracted for total RNA following a typical TRIzol-chloroform protocol. cDNA was synthesized from 1 μg of total RNA using the High-Capacity cDNA Reverse Transcription Kit with RNase inhibitor (Applied Biosystems, Waltham, MA, USA). The cDNA was diluted with nuclease-free water at a 1-to-7 ratio to yield cDNA working solution for qPCR analysis. qPCR was performed using QuantStudio 3 (Applied Biosystems, Waltham, MA, USA) using PowerUp SYBR Green Master Mix (Applied Biosystems, Waltham, MA, USA). The 20 µL reaction consists of 7 µL cDNA, 1.5 µL forward/reverse primers (10 nM), and 10 µL SYBR Green. Relative expression was calculated using the comparative Ct method. The 18SrRNA gene was used as the housekeeping gene to normalize loading variations.

### 2.5. BrdU Cell Proliferation Assay

The proliferative capacity of cells exposed to both iron imbalance and LPS was assessed using a BrdU assay (EMD Millipore Corp., Burlington, MA, USA). BrdU, a thymidine analog, incorporates into the DNA of cells actively undergoing replication during the S phase of the cell cycle. Cells were cultured in 96-well plates and allowed to reach 50–60% confluence prior to treatment, as described in the previous section. At the end of the LPS treatment, BrdU was added to the culture medium at a dilution of 1:2000 and incubated for 6 h. After incubation, cells were treated with a primary anti-BrdU antibody (1:100) for 1 h at room temperature, followed by incubation with a horseradish peroxidase (HRP)-conjugated goat anti-mouse IgG secondary antibody (1:1000) for 30 min at room temperature. Absorbance was measured at dual wavelengths of 450–540 nm. Three control groups were included: negative controls, positive controls, and a group cultured in medium without fetal bovine serum. The assay was performed in three technical replicates using cells from three biological replicates across different passages.

### 2.6. Untargeted Metabolomics

Metabolomics analysis at the West Coast Metabolomic Center at UCD was performed using a protocol previously described [27]. Briefly, treated cells were removed from plates using a cell scraper and centrifuged to form a cell pellet. The cell pellets were rinsed with ice-cold PBS and stored at −80 °C prior to untargeted gas chromatography-mass spectrometry (GC-MS). Details regarding the untargeted metabolomics assay are presented in Supplemental Materials [28,29]. The normalized signal intensities of all measurable free low-molecular-weight metabolites were used to determine primary metabolism.

### 2.7. Statistics

Data were analyzed using one-way or two-way ANOVA analysis with Geisser-Greenhouse correction. Statistical significance was determined at *p* < 0.05. Statistical analyses of untargeted metabolome data were performed using MetaboAnalyst platform (v. 6.0; https://www.metaboanalyst.ca/; accessed on 1 July 2024). Metabolome data (peak intensity profile) were normalized using square root transformation and mean centering prior to univariate or multivariate analyses. One-way ANOVA was used to identified metabolites that were significantly altered by treatment. Unsupervised principal component analysis (PCA) and supervised partial least square discriminant analysis (PLSDA) analyses were conducted for classification and identifying metabolic signatures. Statistical significance was declared at FDR-adjusted *p* (q-value) < 0.1.

## 3. Results

### 3.1. Effects of Iron Excess and Deficiency on Viability and Cellular Iron Status

**Figure 1:**
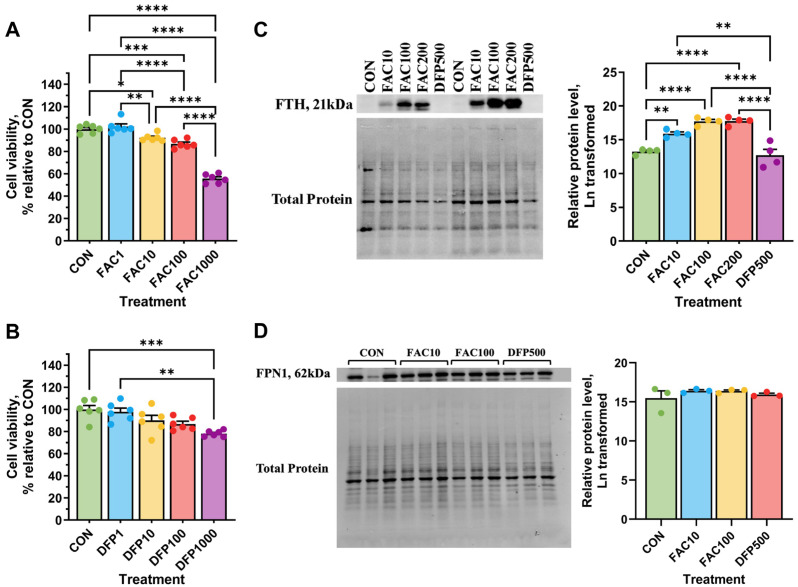
Effects of deferiprone (DFP) and ferric ammonium citrate (FAC) on cell viability and protein expression of H-ferritin and ferrportin in IPEC-J2 cells after 72 h of treatment. (**A**) Cell viability following deferiprone (DFP) treatment at 1, 10, 100, and 1000 µM (*n* = 6/treatment). (**B**) Cell viability following ferric ammonium citrate (FAC) treatment at 1, 10, 100, and 1000 µM (*n* = 6/treatment). (**C**) Representative immunoblot (left, *n* = 2/treatment) and total protein-normalized expression levels of H-ferritin (FTH, right) in response to FAC (10, 100, and 200 µM) and DFP (500 µM) treatment (*n* = 4/treatment). (**D**) Immunoblot (left, *n* = 3/treatment) and total protein-normalized expression levels of ferroportin (FPN1, right) in response to FAC (10 and 100 µM) and DFP (500 µM) treatment (*n* = 3/treatment). Data were analyzed by one-way ANOVA with Tukey’s post hoc test. CON: untreated control; DFP: iron chelator for iron deficiency model; FAC: iron overload model. For significant treatment effect, pairwise differences are indicated as * *p* < 0.05, ** *p* < 0.01, *** *p* < 0.001, **** *p* < 0.0001.

Cell viability was not significantly affected by 1 µM FAC, while treatment with 10 µM and 100 µM FAC resulted in a moderate but significant reduction to 92% and 86%, respectively (*p* < 0.05, Figure 1A). A higher dose of 1000 µM FAC led to a substantial decrease in viability to 55%, indicating cytotoxicity. Increasing DFP concentrations did not significantly affect cell viability until 1000 µM (*p* < 0.05, Figure 1B). However, viability remained above the 80% cytotoxicity threshold under 1000 µM DFP.

To confirm changes in cellular iron stores, we next analyzed ferritin heavy chain (FTH) protein expression in response to subtoxic levels of FAC (10, 100, and 200 µM) and DFP (500 µM, Figure 1C). FTH expression in DFP-treated cells remained low and did not differ from the control (CON). In contrast, FAC treatment at all concentrations significantly increased FTH expression compared to both CON and DFP treatments (*p* < 0.01), indicating that 10 µM of FAC was sufficient to induce cellular iron accumulation. Neither FAC (10 and 100 µM) or DFP (500 µM) significantly affected ferroportin (FPN1) expression (Figure 1D). Based on these findings, 10 µM FAC and 500 µM DFP were selected for subsequent experiments.

### 3.2. Effects of Iron Excess and Deficiency on Iron Transporter Gene Expression

**Figure 2:**
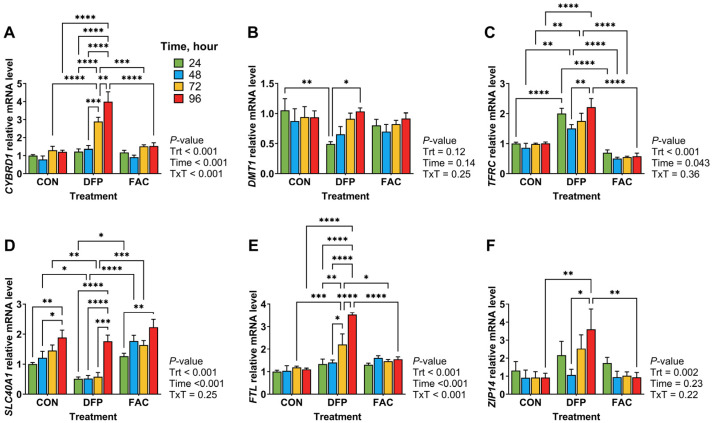
Effects of iron deficiency and overload on iron transporter gene expression in IPEC-J2 cells over 96 h (*n* = 4/treatment). (**A**–**F**) mRNA expression of *CYBRD1*, *DMT1*, *TFRC*, *SLC40A1* (FPN1), *FTL* and *ZIP14*, respectively. Data were analyzed by two-way ANOVA to assess the main effects of treatment, time, and their interaction, followed by Tukey’s post hoc test. CON, untreated control; DFP, deferiprone (500 µM, iron deficiency model); FAC, ferric ammonium citrate (10 µM, iron overload model). *p* values for main effects and interactions are shown alongside each panel. Pairwise differences are indicated as * *p* < 0.05, ** *p* < 0.01, *** *p* < 0.001, **** *p* < 0.0001.

The mRNA expression of genes involved in iron uptake (*CYBRD1*, *DMT1*, *TFRC*, and *ZIP14*) and export (*SLC40A1*) at 24, 48, 72, and 96 h of the treatments is shown in Figure 2. The expression of *CYBRD1* in DFP-treated cells was significantly higher at 72 and 96 h compared to 24 and 48 h, and it was also significantly higher than that in CON and FAC-treated cells at the same time points (*p* < 0.01, Figure 2A). Compared to the control, DFP-treated cells had lower *DMT1* at 24 h (*p* < 0.01, Figure 2B), which gradually increased over time, becoming significantly higher at 96 h compared to 24 h (*p* < 0.05). DFP treatment significantly increased *TFRC* expression at all time points compared to the CON and FAC groups (*p* < 0.01, Figure 2C). *SLC40A1* expression was significantly lower in the DFP group at 48 and 72 h compared to CON and FAC (*p* < 0.05, Figure 2D). Interestingly, all groups exhibited an increase in *SLC40A1* at 96 h post treatment (*p* < 0.05). *FTL* expression in the DFP group was significantly increased at 72 and 96 h (*p* < 0.05, Figure 2E). Additionally, DFP treatment significantly elevated *ZIP14* expression at 96 h in comparison with the other groups (*p* < 0.01, Figure 2F).

### 3.3. Effects of Iron Imbalance and LPS Treatment on Cellular Proliferation and the Expression of Genes Encoding for Iron Regulatory Proteins and Inflammatory Mediators

**Figure 3:**
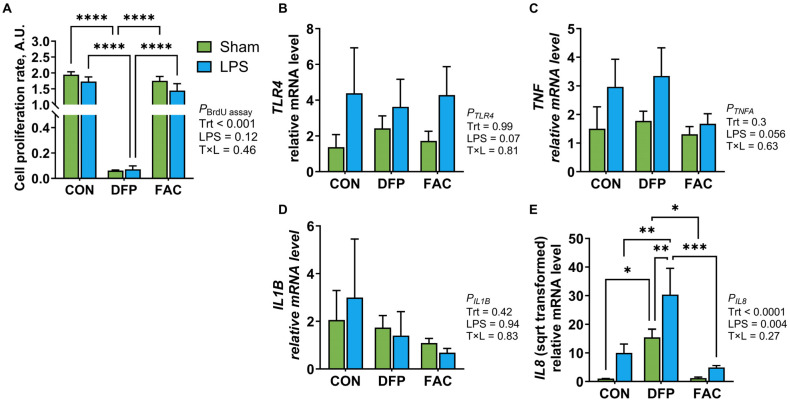
Effects of LPS stimulation on cellular proliferation and the expression of inflammatory mediators in IPEC-J2 cells pretreated with DFP or FAC (*n* = 4/treatment). Cells were pre-exposed to deferiprone (DFP, 500 µM, 48 h) or ferric ammonium citrate (FAC, 10 µM, 48 h), followed by LPS stimulation (1 µg/mL, 4 h). Sterile PBS served as sham exposure. (**A**–**E**) Cellular proliferation and mRNA expression of *TLR4*, *TNF*, *IL1B*, and *IL8* genes, respectively. Data were analyzed by two-way ANOVA to assess the main effects of treatment, LPS, and their interaction, followed by Tukey’s post hoc test. CON, untreated control; DFP, iron deficiency model; FAC, iron overload model. *p* values for main effects and interactions are shown alongside each panel. Pairwise differences are indicated as * *p* < 0.05, ** *p* < 0.01, *** *p* < 0.001, **** *p* < 0.0001.

DFP (500 μM) significantly reduced the rate of DNA replication compared to both the FAC and CON groups at 24 h of treatment (*p* < 0.001, Figure 3A), regardless of LPS presence. In contrast, 10 μM of FAC did not alter cellular proliferation compared to CON. LPS had no significant effect on proliferation under any iron status, with only a numerical reduction in CON and FAC-treated cells.

**Figure 4:**
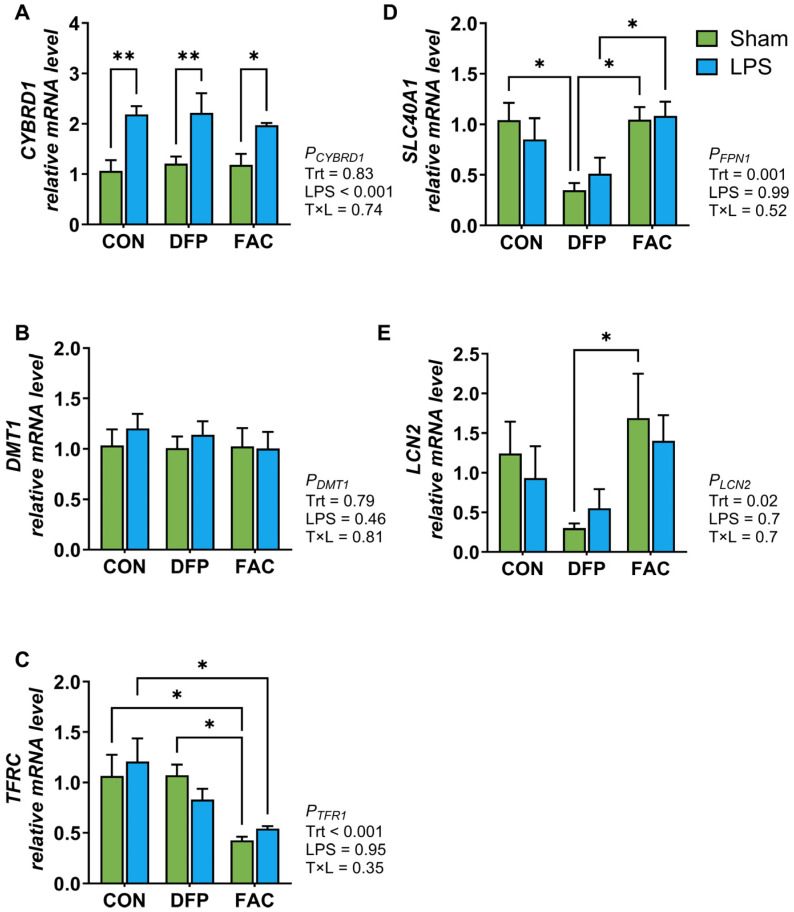
Effects of LPS stimulation on expression of iron regulatory genes in IPEC-J2 cells pretreated with DFP or FAC (*n* = 4/treatment). Cells were pre-exposed to deferiprone (DFP, 500 µM, 48 h) or ferric ammonium citrate (FAC, 10 µM, 48 h), followed by LPS stimulation (1 µg/mL, 4 h). (**A**–**E**) mRNA expression of *CYBRD1*, *DMT1*, *TFRC*, *SLC40A1* (FPN1), and *LCN2* genes, respectively. Data were analyzed by two-way ANOVA to assess the main effects of treatment, LPS, and their interaction, followed by Tukey’s post hoc test. CON, untreated control; DFP, iron deficiency model; FAC, iron overload model. *p* values for main effects and interactions are shown alongside each panel. Pairwise differences are indicated as * *p* < 0.05, ** *p* < 0.01.

We further assessed the interplay between iron imbalance and LPS exposure on expression of genes involved regulating iron homeostasis and inflammation. LPS exposure tended to increase the gene expression of *TLR4* and *TNF* (*p* ≤ 0.07, Figure 3B,C), regardless of cellular iron status, but did not affect *IL1B* expression (Figure 3D). LPS significantly increased *IL8* expression (*p* = 0.004, Figure 3E), whereas DFP treatment elevated *IL8* expression (*p* < 0.0001) independent of LPS exposure. The LPS treatment significantly increased *CYBRD1* expression, irrespective of iron availability (*p* < 0.05, Figure 4A), but did not affect the expression of *DMT1*, *TFRC*, *SLC40A1*, or *LCN2* (Figure 4B–E). Treatment with FAC reduced *TFRC* expression compared to the CON group (*p* < 0.05). *SLC40A1* expression was significantly lower in the DFP group compared to both the CON and FAC groups (*p* < 0.05) in the absence of LPS. *LCN2* expression was lower in DFP-treated cells, with a significant difference detected between DFP and FAC groups under the sham (sterile PBS) treatment (*p* < 0.05).

### 3.4. Metabolic Responses to Iron Excess, Deficiency, and Restoration After Deprivation

**Figure 5:**
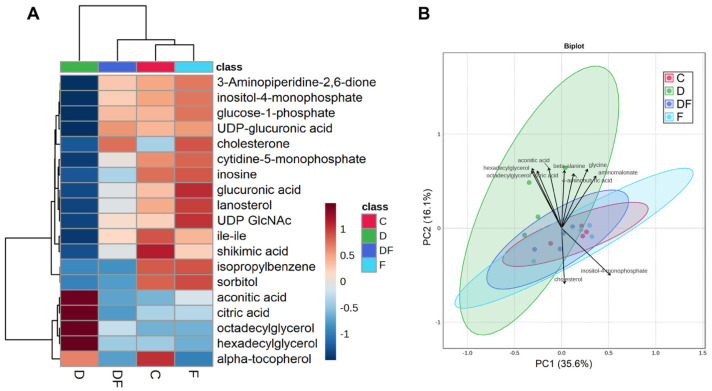
Unsupervised analysis of untargeted metabolomics in IPEC-J2 cells under iron deficiency, overload, and repletion (*n* = 4/treatment). Cells were left untreated (control, C) or treated with deferiprone (D, 500 µM, 48 h), ferric ammonium citrate (F, 10 µM, 48 h), or sequentially (DF) with deferiprone (500 µM, 24 h) followed by ferric ammonium citrate (10 µM, 24 h). (**A**) Heatmap of 19 identified metabolites significantly affected by treatment (*q* < 0.1, see Table 1). (**B**) Principal component analysis (PCA) biplot.

**Figure 6:**
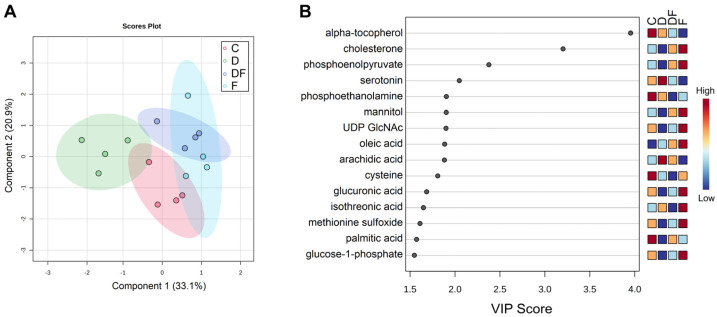
Supervised analysis of untargeted metabolomics in IPEC-J2 cells under iron deficiency, overload, and repletion (*n* = 4/treatment). Cells were left untreated (control, C) or treated with deferiprone (D, 500 µM, 48 h), ferric ammonium citrate (F, 10 µM, 48 h), or sequentially (DF) with deferiprone (500 µM, 24 h) followed by ferric ammonium citrate (10 µM, 24 h). (**A**) 2D plot of Partial Least Squares Discriminant Analysis (PLS-DA). (**B**) Variable Importance in Projection (VIP) score plot identifying the most influential metabolites in the PLS-DA model.

A total of 488 metabolites were detected, of which 129 were identified in the reference library. Treatment significantly altered 71 metabolites (FDR < 0.1), with 19 of these identified in the library (Table 1 and Table S3) and visualized in a heatmap (Figure 5A and Figure S1). Hierarchical analysis based on the 19 altered metabolites revealed the most distinct profile in the iron deficiency (D) group and the greatest similarity between the control (C) and iron excess (F) treatment. Principal component analysis corroborated this finding, showing greater separation of D mainly along the PC2 (*y*-axis) from the other groups (Figure 5B). In comparison with the C and F treatments, 13 out of 19 metabolites were significantly decreased by the D treatment, while 4 were significantly increased (Table 1). DF treatment partially or completely restored these metabolites to levels that were not statistically different from C treatment, except inosine, isopropylbenzene, and sorbitol, which were not changed by iron repletion. Cholesterone was significantly lower in the C and D treatments compared to the DF and F treatments, whereas alpha-tocopherol exhibited the opposite pattern, being significantly higher in C and D compared to DF and F. The PCA biplot revealed key metabolites that contributed to the observed distribution pattern. A group of 8 metabolites, including aconitic acid, citric acid, hexadecylglyderol, hexadecylglycerol, 4-aminobutyric acid, and beta-alanine, contributed the most to separating the D group samples from those of the other groups. In contrast, inositol-4-monophosphate and cholesterol were signature metabolites that distinguished the other groups from the D group. The 2D plot from the supervised PLSDA showed a clearer separation between groups (Figure 6A) and identified alpha-tocopherol and cholesterone as the top contributors to sample clustering by treatments. These were followed by phosphoenolpyruvate and serotonin, all of which had a VIP scores greater than 2 (Figure 6B).

## 4. Discussion

Enterocytes in the proximal small intestine are essential for nutrient absorption and serve as physical barrier against environmental pathogens and insults [11,30]. This study employed non-transformed IPEC-J2 cells derived from the jejunum of neonatal piglets to investigate intestinal epithelial responses to iron deficiency (ID) and excess (IE), as well as the role of iron imbalance in regulating cellular metabolism and inflammatory responses.

### 4.1. Effects of Iron Excess and Deficiency on Cell Viability and Iron Metabolism

Given that the XTT assay measures mitochondrial enzyme activity, reductions in cell viability at 1000 µM DFP and FAC at any tested concentrations indicated that both ID and IE impaired mitochondrial metabolic activity. DFP, an FDA-approved iron chelator utilized for the treatment of iron overload, preferentially removes intracellular labile iron [31]. As such, DFP may also chelate iron from the active sites of enzymes (e.g., succinate dehydrogenase), thereby impairing mitochondrial metabolism. Conversely, iron overload may play a role in the disruption of mitochondrial metabolism by inducing oxidative stress via the Fenton reaction. At all FAC concentrations in the current study, H-ferritin was significantly upregulated, confirming successful induction of cellular iron accumulation. In contrast, H-ferritin was nearly undetectable in some cells treated with 500 µM DFP.

The time-course analysis revealed transcriptional changes of iron regulatory genes in response to ID and IE. Most of these genes are primarily regulated by iron regulatory proteins (IRPs) and hypoxia-inducible factors (HIFs), of which mechanisms have been extensively reviewed elsewhere [32,33,34]. DMT1, located on the brush border of the intestinal epithelium, mediates dietary iron uptake [35], while TFRC imports transferrin-bound iron across basolateral membrane. The presence of IREs in the 3′UTRs of both genes allows their mRNA stability to be inversely regulated by cellular iron levels through modulation of IRP binding activity [36]. Indeed, *TFRC* expression was highest in DFP-treated cells and lowest in FAC-treated cells at all time points, serving as a mechanism to prevent excessive iron uptake. This pattern aligns with previous reports showing reduced *TFRC* expression in the duodenal mucosa of piglets fed high-iron diets [37,38]. In contrast, the overall *DMT1* expression was not significantly affected by iron status, although a gradual increase was observed in DFP-treated cells over time. Similarly, Deng et al. (2024) reported that iron dextran supplementation did not alter *DMT1* mRNA or protein expression in the small intestine of piglets or IPEC-J2 cells [39]. In rodent models and Caco-2 cells, however, the IRE+ isoform of *DMT1* is negatively regulated by iron treatment [40,41,42]. This discrepancy may be due to the expression of non-IRE-containing *DMT1* isoforms, which have been identified in both rodents and humans, but not yet in pigs [43,44].

While *SLC40A1* and *FTL* are post-transcriptionally regulated by IRPs via binding to their 5′IREs, their expressions, along with *CYBRD1*, are also regulated by transcription factors such as nuclear factor erythroid 2-related factor 2 (NRF2) and HIFs, which are impacted by cellular iron status [45,46,47]. Under hypoxia, HIF activation promotes the expression of *CYBRD1*, *DMT1* and *SLC40A1* to enhance iron absorption and erythropoiesis [33,34]. Iron deficiency can induce HIFs by inhibiting HIF prolyl hydroxylase, which is an iron-requiring enzyme that hydroxylates HIFs for degradation. Therefore, the upregulation of *CYBRD1*, *DMT1*, and *SLC40A1* over time in DFP-treated cells probably reflects HIF-mediated transcriptional activation. Additionally, NRF2 is known to protect cells from oxidative stress induced by elevated intracellular labile iron by upregulating ferritin genes (*FTH* and *FTL*) to enhance iron storage and *SLC40A1* expression to facilitate iron export [47]. However, this mechanism does not completely explain the expression pattern observed here. Given the complexity of these regulatory networks, we cannot definitively attribute the observed changes to specific mechanisms. Notably, DFP-induced ID elicited the most dynamic gene expression responses over time, highlighting its significant impact on iron homeostasis. The transcriptional changes observed in this study await validation at the protein level in future work to better understand iron regulation under ID and IE conditions.

### 4.2. Iron Imbalance Has Limited Effects on the Inflammatory Response

In contrast to XTT assay results, which measures mitochondrial enzyme activities, findings from the BrdU assay suggest that iron restriction disrupts DNA replication, possibly at the S phase of a cell cycle, and results in growth arrest despite minimal influence on metabolic rate. This aligns with studies showing antiproliferative effects of DFP and other iron chelators in tumor cell lines [48,49,50].

IPEC-J2 cells express most toll-like receptors and a spectrum of cytokines and exhibit inflammatory response to immune stimuli [51]. However, in this study, the findings of marginal upregulation of *TLR4* and *TNF* (<4 folds, *p* ≤ 0.07) and unaffected *IL1B* expression after 4 h LPS exposure does not support a strong inflammatory property of IPEC-J2 cells. In contrast, the robust induction of *IL8*, a chemokine that attracts neutrophils, after LPS exposure suggests a more prominent role of gut epithelial cell in immune cell recruitment rather than direct inflammatory response. Although cellular iron status was shown to modulate LPS-induced inflammatory responses in porcine alveolar macrophage cells [19], the lack of significant changes in inflammatory genes under ID or IE suggests a limited role of iron in modulating inflammatory signaling in gut epithelial cells. The significant induction of *IL8* by ID has not been previously reported. Although the underlying mechanism is unclear, hypoxia-response element sequence have been identified in IL8 promoter, and transcriptional activation by HIF has been confirmed in human bone marrow stromal cells and ovarian carcinoma cells [52,53]. It is therefore plausible that ID upregulates IL8 expression through HIF activation.

For iron metabolism-related genes, only *CYBRD1,* a membrane-bound ferrireductase, showed a significant response to LPS, exhibiting upregulated expression. This finding diverges from previous work demonstrating the downregulation of both mRNA and protein expression of CYBRD1 (DCYTB), DMT1 (IRE+ isoform), and SLC40A1 in the duodenum of rats 6 h after LPS injection, which resulted in acute hypoferremia partly through reducing intestinal iron absorption [54]. During inflammation, the down-regulation of *CYBRD1* reduces the conversion of ferric to ferrous iron, thereby limiting uptake by DMT1 and promoting the hypoferremic response. The upregulation of *CYBRD1* observed in the current study may reflect the absence of inflammation-induced systemic hepcidin signaling. Previously, it has been shown that low levels of hepcidin results in marked upregulation of *CYBRD1* [55]. Because CYBRD1 lacks an IRE, its transcription is primarily regulated by HIF-2α, which in turn has been shown to be regulated by systemic hepcidin levels [56]. Thus, in the absence of hepcidin-mediated HIF-2α suppression, the expression of *CYBRD1* may not follow the typical down-regulation associated with the hypoferremic response. The absence of a coordinated response in IPEC-J2 cells suggests limitations in this model to replicate systemic iron-inflammation crosstalk in intestine.

### 4.3. Effects of Iron Imbalance on Intermediary Metabolism

DFP-induced ID led to the most profound changes in intermediary metabolism. The main metabolic features detected were elevated levels of TCA intermediates (citric acid, aconitic acid) and alkylglycerols (octadecylglycerol, hexadecylglycerol), along with reduced levels of metabolites involved in glucuronic acid synthesis (glucose-1-phosphate, UDP-glucuronic acid, glucuronic acid), cholesterol metabolism (lanosterol, cholesterone), and nucleotide synthesis (inosine and cytidine-5-monophosphate). Under ID conditions, aconitase carries out its role as IRP1 instead of its enzymatic function in TCA cycle. Therefore, accumulation of citric acid and aconitic acid in DFP-treated cells likely reflect disruptions in the TCA cycle rather than increased metabolic flux. Similar findings were observed in DFP-treated porcine alveolar macrophage cells [19]. Both glucuronic acid and UDP-GlcNAc can be synthesized de novo from glucose [57]. Therefore, the concomitant reduction in these metabolites and disrupted TCA cycle indicates a significant shift in glucose metabolism from aerobic respiration toward anaerobic glycolysis for rapid energy production. Oexle et al. (1999) also observed that exposure to the iron chelator, desferrioxamine, suppressed multiple TCA cycle enzymes while enhancing lactate production in human erythroleukemia cell line, consistent with a metabolic switch toward glycolysis [58]. Importantly, the metabolic changes induced by DFP treatment were partially or completely reversed with iron supplementation in DF-treated cells, underscoring metabolic resilience of enterocytes in withstanding transient chelator-induced ID.

The reduction in cytidine-5-monophosphate in DFP-treated cells suggests impaired nucleotide synthesis, consistent with suppressed BrdU incorporation observed in the cell proliferation assay. Furthermore, octadecylglycerol and hexadecylglycerol are precursors for synthesis of alkenyl ether lipid such as plasmalogens, which exhibit antioxidant properties and play crucial roles in maintaining cell membrane integrity, facilitating cell signaling and cholesterol esterification [59,60]. Plasmalogens have been implicated in protection against ferroptosis, an iron-dependent, non-apoptotic form of cell death characterized by excessive lipid peroxidation, although evidence remains mixed [61,62,63]. Given the rapid turnover of intestinal epithelial cells, reductions in cellular proliferation and alterations in membrane integrity during iron-deficient conditions may have broad physiological consequences, including impaired intestinal barrier function and reduced nutrient absorption [64]. Despite these impacts, however, the metabolic changes observed highlight shifts towards increased energy production and the conservation of resources within the cell, consistent with the transcriptional responses observed in the iron chelator-treated group in which intracellular iron preservation was promoted through alterations in the expression of regulatory genes such as *TFRC* and *CYBRD1*. Together, these adaptations suggest that enterocytes reprogram metabolic activity to maintain critical biological functions during iron deficiency.

Although lipid peroxidation and other ferroptosis markers were not directly analyzed in this study, we observed a significant reduction in alpha-tocopherol, the primary form of vitamin E, in both DF and F groups that received excess iron supplementation. Alpha-tocopherol is a critical lipophilic antioxidant that scavenges intracellular free radicals and protects lipoproteins and polyunsaturated fatty acids from oxidative damage [65,66]. Supplementation of alpha-tocopherol was shown to alleviate lipid peroxidation and rescue Gpx4-deficient hematopoietic stem and progenitor cells from ferroptosis in vitro [67]. Similar protective effects were also observed in a mouse model of necrotizing enterocolitis, where vitamin E treatment alleviated intestinal injury and inflammation through preventing lipid peroxidation and inhibiting ferroptosis in regulatory T cells [68]. Because of prolonged sample storage, oxidative stress markers cannot be accurately determined in the current study. Future studies are warranted to confirm whether iron overload leads to increased oxidative damage.

Notably, alpha-tocopherol and cholesterone were identified as the top two metabolic features that distinguished treatment groups in the supervised analysis (PLS-DA). This finding aligns with our previous work showing that iron overload increased cellular cholesterol in porcine alveolar macrophages [19]. Furthermore, it has been shown that treating human umbilical vein endothelial cells (HUVECs) with excess iron upregulates genes involved in the cholesterol synthesis pathway. This response was thought to be mediated by sterol regulatory element-binding protein 2 (SREBP2), a transcription factor that regulates cholesterol synthesis, which was upregulated as a result of the iron loading [69]. Together, our findings suggest that iron-induced cholesterol biosynthesis and increased utilization of alpha-tocopherol may represent a coordinated cellular response to both metabolic and possible oxidative stress induced by iron loading in gut epithelial cells. Additionally, these findings may represent a basis for the minimal transcriptional changes observed in the iron-supplemented groups despite increased ferritin levels and suggests a potential mechanism for resilience against iron loading.

## 5. Conclusions

This study demonstrated that iron imbalance significantly influences cellular metabolism and gene expression in IPEC-J2 cells, with ID exerting the most pronounced effects. Iron deficiency-impaired cell proliferation suppressed cholesterol biosynthesis, and disrupted the TCA cycle, triggering a metabolic shift toward glycolysis for energy production. Among the metabolites significantly altered by ID, most of them were partially or fully reversed upon iron restoration (DF). In contrast, IE upregulated ferritin expression, depleted antioxidants (vitamin E), and enhanced cholesterol biosynthesis. Despite these metabolic changes, iron imbalance had marginal impacts on inflammatory signaling under short-term LPS exposure. Together, these findings highlight the metabolic plasticity of IPEC-J2 cells to altered cellular iron levels and underscore the potential protective role of vitamin E against iron overload. This study, however, is limited by a small sample size and the use of a single cell type on a 2D monoculture model and reveals some discrepancies when compared with findings from in vivo studies. Moreover, our analysis was limited to the evaluation of transcriptional changes in genes related to iron metabolism and inflammation. Future studies comparing multiple epithelial cell lines and incorporating translational and functional assessments—such as protein expression, barrier permeability, and oxidative stress markers—are warranted to provide a more comprehensive understanding of how iron imbalance regulates gut epithelial function.

## Figures and Tables

**Table 1:** Peak intensity of metabolites significantly affected by treatment (*q*-value < 0.1).

Metabolite	C ^1^		D		DF		F		*q*-Value
	Mean	SEM	Mean	SEM	Mean	SEM	Mean	SEM	
3-Aminopiperidine-2,6-dione	3124.8 ^a^	299.4	1430.8 ^b^	161.6	2903.8 ^a^	299.1	3502.8 ^a^	524.6	0.013
aconitic acid	524.8 ^b^	72.5	3322.3 ^a^	964.7	496.0 ^b^	103.8	797.3 ^b^	141.6	0.006
cholesterone	921.0 ^b^	177.3	533.8 ^c^	104.8	1706.3 ^a^	110.3	1874.0 ^a^	274.4	0.012
citric acid ^2^	154,670.3 ^b^	13,419.7	1,408,826.8 ^a^	357,909.6	101,176.8 ^b^	20,492.3	164,589.5 ^b^	11,606.3	<0.001
cytidine-5-monophosphate	42,332.5 ^a^	6691.8	6888.8 ^b^	2799.4	27,693.0 ^a^	8910.2	53,602.0 ^a^	11,961.3	0.017
glucose-1-phosphate	10,421.5 ^a^	1378.1	3750.5 ^b^	980.5	10,443.5 ^a^	1644.5	12,552.3 ^a^	1133.7	0.017
glucuronic acid	1057.3 ^a^	166.9	619.5 ^b^	74.5	915.8 ^ab^	107.6	1320.3 ^a^	186	0.097
hexadecylglycerol	1249.3 ^b^	136.5	7011.5 ^a^	2372.2	1230.3 ^b^	28.3	1072.3 ^b^	130.2	0.012
ile-ile	2824.0 ^a^	609.7	783.5 ^b^	261.2	1716.0 ^a^	155.9	2098.8 ^a^	450.3	0.097
inosine	1023.8 ^a^	161.6	298.3 ^b^	51.2	559.3 ^b^	147	1082.0 ^a^	117.4	0.017
inositol-4-monophosphate^2^	27,104.5 ^a^	2906.9	7251.5 ^b^	1097.1	22,921.5 ^a^	2277.6	31,200.5 ^a^	2461	<0.001
isopropylbenzene	15,517.3 ^a^	1345.4	10,806.0 ^b^	920.1	11,053.0 ^b^	1357.7	15,495.3 ^a^	628.4	0.097
lanosterol	614.3 ^a^	89.7	244.8 ^b^	55.2	463.8 ^a^	73.2	853.0 ^a^	166.8	0.070
octadecylglycerol	929.5 ^b^	111.6	7322.8 ^a^	2267.1	1554.8 ^b^	581.5	922.5 ^b^	110.4	0.019
shikimic acid	7532.8 ^a^	851.5	2443.5 ^b^	593.5	4236.8 ^ab^	653.7	5393.3 ^a^	1569.2	0.097
sorbitol	37,957.0 ^a^	4338.3	18,056.8 ^b^	4348.2	18,732.0 ^b^	3177.9	40,249.3 ^a^	5834.5	0.088
tocopherol alpha-	3966.0 ^a^	134	3717.8 ^a^	356.8	2778.0 ^b^	78.3	2687.3 ^b^	228.2	0.022
UDP GlcNAc	2566.0 ^a^	691.5	1268.3 ^b^	149.2	2322.5 ^a^	367.4	3286.3 ^a^	348.6	0.097
UDP-glucuronic acid ^2^	5516.3 ^a^	731.1	1331.5 ^b^	6.3	6296.3 ^a^	825.6	6201.0 ^a^	761.4	<0.001

^1^ C: iron-replete control, D: iron deficiency (deferiprone treatment), DF: iron restoration from deficiency, F: iron excess. ^2^ *q*-values are reported to three decimal places. Approximate value (<0.001) is presented if the *q*-value is less than 0.001. ^a, ab, b, c^ Values that share no common letters are significantly different (*p* < 0.05).

## Data Availability

All data have been reported here and in the Supplemental Materials.

## Supplementary Materials

The following supporting information can be downloaded at: https://www.mdpi.com/article/10.3390/metabo15110691/s1. Supplemental Methods; Table S1: Primer sequences for tested genes; Table S2: Effects of iron deficiency, restoration, and excess on relative fold change of metabolites compared to the iron-replete control; Table S3: Retention indices and quantitative *m*/*z* values of significantly altered metabolites; Figure S1: Heatmap of 19 identified metabolites significantly affected by treatment.

## Abbreviations

The following abbreviations are used in this manuscript:

BrdU	Bromodeoxyuridine
CYBRD1	Duodenal Cytochrome B
DFP	Deferiprone
DMT1	Divalent Metal Transporter 1
FAC	Ferric Ammonium Citrate
FPN1	Ferroportin
FTH	Ferritin Heavy Chain
FTL	Ferritin Light Chain
HIF	Hypoxia-Inducible Factor
ID	Iron Deficiency
IE	Iron Excess
IL8	C-X-C motif chemokine ligand 8, CXCL8
IRP	Iron Regulatory Protein
LCN2	Lipocalin-2
LPS	Lipopolysaccharide
PCA	Principle Components Analysis
PLSDA	Partial Least Squares Discriminant Analysis
ROS	Reactive Oxidative Species
SLC40A1	Solute carrier family 40 member 1 (Ferroportin)
TFRC	Transferrin receptor 1
TLR4	Toll like Receptor 4
TNF	Tumor Necrosis Factor alpha
UTR	Untranslated Region
VIP	Variable Importance in Projection
ZIP14	Solute carrier family 39 member 14
