# Supplementary material for: Iron Stress Reprograms Enterocyte Metabolism
Source: Metabolites. 2025 Oct 24;15(11):691. doi: 10.3390/metabo15110691 (PMC12654117; doi:10.3390/metabo15110691)
Supplement: Supplementary file 1 [file metabolites-15-00691-s001.zip › metabolites-3931252-supplementary.pdf]

Supplemental Methods: Primary Metabolite Analysis

Metabolite profiling of treated IPEC-J2 cells was completed at the University of California, Davis Genome Center's West Coast Metabolomics Center. Samples were analyzed for primary metabolism using a 7890A gas chromatograph coupled to a LECO time-of-flight mass spectrometer (GC-TOF-MS).

Derivatization: Metabolites were extracted using 1mL of 3:3:2 ACN:IPA:H2O (v/v/v). Half of each sample was dried to completeness then derivatized using 10 uL of 40 mg/mL of methoxyamine in pyridine. After being shaken at 30˚C for 1.5 hours, 91 uL of MSTFA + FAMEs was added to each sample and was shaken again at 37˚C for 30 minutes to finish derivatization.

GC-Conditions: 0.5 uL of derivatized sample was injected using a splitless method onto a RESTEK RTX-5SIL MS column (30 m length x 0.25 mm internal diameter with 0.25 µm film made of 95% dimethyl/5%diphenylpolysiloxane) with an Intergra-Guard at 275˚C with a helium flow of 1 mL/min. The GC oven temperature was set to hold at 50˚C for 1 min then raised 20˚C/minute until 330˚C and held for 5 min. The transfer line was set to 280˚C while the EI ion source was set to 250˚C. The mass spec parameters collected data from 85m/z to 500m/z at an acquisition rate of 17 spectra/sec. Fatty acid methyl esters (FAME) were utilized as internal standards to check instrument response in each sample. Method blanks, external quality controls, and sample pools were injected every 10 samples. Additional chromatographic details can be found at Fiehn et al. (2008) [28]

Data Processing: Raw data files were pre-processed using ChromaTOF vs. 2.32 without smoothing, 3 s peak width, baseline subtraction just above the noise level, and automatic mass spectral deconvolution and peak detection at signal/noise levels of 5:1 throughout the chromatogram. Apex masses were reported for use in the BinBase algorithm [29]. Result files were exported to a data server with absolute spectra intensities and further processed by a filtering algorithm implemented in the metabolomics BinBase database. Spectra were matched to the BinBase database based on retention index, unique ion presence, and mass spectral similarity, with an isomer filter applied. Quantification was reported as peak height with the unique ion as default. Compounds that were positively detected in >10% of samples were included in the final report. Raw results were normalized using a variant of a vector normalization. First, the sum of all peak heights, the mTIC, for all identified metabolites (excluding unknowns) in each sample were calculated. Because the mTIC averages were not found to be significantly different between treatment groups, the data were then normalized to the total average mTIC of the samples.

Table S1. Primer Sequences for Tested Genes

Gene Symbol	Gene Name	Forward primer 5′-3′	Reverse primer 5′-3′
CYBRD1	Duodenal Cytochrome B	AGATTGGCCCTGGAG ACTGA	CAAGGAAGCCTTGGGTG AAG
DMT1 (SLC11A2)	Solute Carrier Family 11 Member 2	AGGATCTAGGGCATGT GGTG	CCACAGTCCAGGAAGGA CAT
FTL	Ferritin Light Chain	AAAACCCAGGACGCT ATGGA	CCAGGAAGTGGTTCTCC AGG
IL1B	Interleukin-1 beta	CCTTGAAACGTGCAAT GATG	TTCAAGTCCCCTGTGAGG AG
IL8 (CXCL8)	CXC motif chemokine ligand 8	GACCCCAAGGAAAAG TGGGT	GAGGCAAGAAGACTTGT GAATGC
LCN2	Lipocalin-2	AGCCAGTTCGCCATAG TATTC	AGCGGACAAAGTTCTCC TTC
RN18S	18S ribosomal RNA	AGGAAAGCAGACATC GACCT	ACCTGGCTGTACTTCCCA TC
SLC40A1 (FPN1)	Solute Carrier Family 40 Member 1 (Ferroportin)	AACGTGGCTTTTACGT GGCT	CAACAAGTCCAAGGGGC TTC
TFRC	Transferrin Receptor 1	GTTTAGCGCAGGAGTG AGGC	TGGGACAAGCAACAGAG GAA
TLR4	Toll like Receptor 4	TCAGTTCTCACCTTCC TCCTG	GTTCATTCCTCACCCAGT CTTC
TNFA	Tumour Necrosis Factor alpha	TGGCCCCTTGAGCATC A	CGGGCTTATCTGAGGTTT GAGA
ZIP14 (SLC39A14)	Solute Carrier Family 39 Member 14	CGAGGAGAACAAGAA GACGG	ACTCACCCTTCCTGGTGA TG

Table S2. Effects of iron deficiency, restoration, and excess on relative fold change of metabolites compared to the iron-replete control (C).

Metabolite	D	DF	F	C	q-value
Amino Acid Metabolism					
alanine	0.72	0.71	1.07	1	0.7885
asparagine	0.75	0.77	0.98	1	0.375
aspartic acid	1.07	0.62	1.08	1	0.7515
beta-alanine	2.22	0.97	1.06	1	0.4377
citrulline	0.83	0.67	1.02	1	0.6193
creatinine	1.17	0.62	1.05	1	0.9438
cysteine	0.62	0.3	0.71	1	0.1299
glutamic acid	0.69	0.84	1.08	1	0.4907
glutamine	0.7	0.76	1.37	1	0.9369
glycine	1.45	0.69	1.09	1	0.4377
glycyl tyrosine	0.31	0.55	0.65	1	0.1037
ile-ile	0.28	0.61	0.74	1	0.0965
isoleucine	0.9	0.85	0.94	1	0.941
leucine	0.64	0.85	0.94	1	0.2845
lysine	0.64	0.63	0.9	1	0.7426
methionine	0.56	0.59	0.82	1	0.26
methionine sulfoxide	0.82	0.98	1.3	1	0.4973
ornithine	0.7	0.93	1.09	1	0.6574
oxoproline	0.66	0.85	1.14	1	0.4273
phenylalanine	0.52	0.6	0.91	1	0.3113
proline	0.89	0.66	1.06	1	0.7515
putrescine	0.94	1.05	1	1	0.999
serine	0.71	0.55	0.8	1	0.7515
spermidine	1.82	1.22	0.9	1	0.8532
threonine	0.88	0.61	1.13	1	0.4653
trans-4-hydroxyproline	1.07	0.91	0.99	1	0.999
tryptophan	0.49	0.58	0.83	1	0.4907
tyrosine	0.57	0.61	0.88	1	0.4551
urea	0.87	1.06	0.99	1	0.8622
valine	0.84	0.89	0.93	1	0.9399
Carbohydrate Metabolism					
1-kestose	0.92	1.08	1.06	1	0.9399
erythritol	0.62	0.84	0.93	1	0.4907
fructose	0.91	0.87	1.04	1	0.9274
fructose-6-phosphate	0.41	0.82	0.94	1	0.4325
galactinol	0.77	0.84	0.89	1	0.999
galactonic acid	1.2	0.8	1.21	1	0.7426
galactose	1.03	1.24	0.97	1	0.8926
galacturonic acid	0.72	0.72	1	1	0.7515
gluconic acid	0.85	0.8	1	1	0.9274
glucose	0.93	1.22	0.98	1	0.7625
glucose-1-phosphate	0.36	1	1.2	1	0.0171

glucose-6-phosphate	0.44	0.79	0.94	1	0.4551
glucuronic acid	0.59	0.87	1.25	1	0.0965
inositol-4-monophosphate	0.27	0.85	1.15	1	0.0002
isomaltose	0.92	1.09	1.21	1	0.9068
maltose	0.84	1.11	0.99	1	0.9369
maltotriose	1.1	1.27	1.08	1	0.9274
mannitol	0.69	1.01	1.27	1	0.4551
mannose	0.43	0.78	0.9	1	0.2634
melibiose	0.72	0.92	1.3	1	0.4551
palatinitol	0.67	0.64	1.08	1	0.2558
raffinose	1.13	1.14	1.16	1	0.9923
ribose	0.84	0.88	0.96	1	0.9839
sorbitol	0.48	0.49	1.06	1	0.0878
UDP-glucuronic acid	0.24	1.14	1.12	1	< 0.0001
xylitol	0.9	0.84	0.93	1	0.8254
xylose	0.87	0.9	0.82	1	0.9274
Energy/Phosphate Transfer					
ethanol phosphate	0.82	0.9	0.91	1	0.9399
ethanolamine	0.93	0.97	0.91	1	0.9438
methanolphosphate	0.53	0.82	0.95	1	0.1959
phosphate	0.82	1.1	0.85	1	0.4551
phosphoethanolamine	0.96	0.8	0.8	1	0.7398
pyrophosphate	0.83	0.83	1.11	1	0.9616
UDP GlcNAc	0.49	0.91	1.28	1	0.0965
Lipid Metabolism					
1-hexadecanol	1.15	0.75	1.05	1	0.6418
1-monopalmitin	1.08	1.08	1.03	1	0.8272
1-monostearin	1.14	1.15	1.05	1	0.6938
2-monoolein	0.44	0.77	0.89	1	0.1959
2-monopalmitin	1.2	1.22	0.93	1	0.3113
adenosine-5-monophosphate	0.39	0.55	1.1	1	0.2704
arachidic acid	1.34	1.01	0.86	1	0.1677
arachidonic acid	0.88	1.13	1.01	1	0.7231
azelaic acid	0.49	0.67	1.22	1	0.1959
beta sitosterol	0.81	0.43	0.79	1	0.765
beta-glycerolphosphate	1.24	0.91	1.09	1	0.9616
cholesterol	0.85	0.91	1.06	1	0.3113
cholesterone	0.58	1.85	2.03	1	0.0124
cis-gondoic acid	0.61	0.8	0.92	1	0.4907
cytidine-5-monophosphate	0.16	0.65	1.27	1	0.0171
D-erythro-sphingosine	0.73	0.74	0.86	1	0.9274
dihydrocholesterol	1.02	1.31	0.94	1	0.4377
glycerol-alpha-phosphate	0.85	1.01	0.99	1	0.9643
heptadecanoic acid	0.8	1.03	0.91	1	0.4907
hexadecylglycerol	5.61	0.98	0.86	1	0.0124
lanosterol	0.4	0.75	1.39	1	0.0698
lauric acid	0.91	0.95	0.98	1	0.7885

linoleic acid	0.69	1	0.94	1	0.6418
monoheptadecanoyl glyceride	1.12	1.15	1	1	0.5502
monomyristin	1.13	1.15	1.02	1	0.4907
myristic acid	0.86	0.93	0.97	1	0.7515
nonadecanoic acid	0.94	0.98	1.02	1	0.999
octadecylglycerol	7.88	1.67	0.99	1	0.019
oleamide	0.86	0.84	0.85	1	0.9274
oleic acid	1.71	2.74	2.16	1	0.7515
palmitic acid	0.81	0.88	0.83	1	0.5502
palmitoleic acid	0.52	0.99	1.05	1	0.3478
squalene	0.79	0.82	1.07	1	0.6755
Nucleotide Metabolism					
5'-deoxy-5'-methylthioadenosine	0.58	0.96	1.28	1	0.3718
adenine	0.61	0.73	1.16	1	0.7515
adenosine	0.31	0.62	0.67	1	0.2558
hypoxanthine	0.31	0.47	0.92	1	0.1299
inosine	0.29	0.55	1.06	1	0.0169
uracil	0.62	0.91	0.93	1	0.4377
Secondary Metabolites or Neurotransmitters					
2,5-dihydroxypyrazine	0.94	0.75	1.11	1	0.941
2-aminobutyric acid	0.51	1.15	0.99	1	0.1191
4-aminobutyric acid	3.41	1.49	1.5	1	0.3376
aminomalonate	1.2	0.5	0.92	1	0.4551
dehydroabietic acid	1.28	0.85	0.96	1	0.7885
noradrenaline	0.44	0.59	0.72	1	0.2918
serotonin	1.18	0.91	0.82	1	0.4551
shikimic acid	0.32	0.56	0.72	1	0.0965
sinigrin	0.65	0.51	0.97	1	0.4273
TCA/Glycolysis					
3-phosphoglycerate	0.15	0.26	0.99	1	0.3711
aconitic acid	6.33	0.95	1.52	1	0.0056
citric acid	9.11	0.65	1.06	1	< 0.0001
fumaric acid	0.41	0.98	1.12	1	0.4038
glyceric acid	0.67	0.77	1.07	1	0.5415
lactic acid	0.98	0.94	1.15	1	0.999
malic acid	0.43	0.9	1.13	1	0.3897
phosphoenolpyruvate	0.8	0.97	1.79	1	0.4551
pyruvic acid	0.62	0.76	0.88	1	0.8532
succinic acid	0.71	0.97	1.01	1	0.9274
Vitamin Metabolism					
dehydrocholecalciferol	0.05	0.69	1.14	1	0.3376
pantothenic acid	0.95	0.84	1.1	1	0.7515
tocopherol alpha-	0.94	0.7	0.68	1	0.0224
Uncategorized/Others					
3-Aminopiperidine-2,6-dione	0.46	0.93	1.12	1	0.0125
isopropylbenzene	0.7	0.71	1	1	0.0965
isothreonic acid	1.12	0.99	1.23	1	0.5502

propyleneglycol	1.36	1.66	1.16	1	0.8711

D: iron deficiency, DF: iron restoration after deficiency, F: iron excess, C: iron-replete control. Data present group mean

q-values are reported for 4 digits after decimal points. For the

Table S3. Retention indices and quantitative m/z values of significantly altered metabolites.

BinBase name	Retention Index	Quantitative m/z
3-Aminopiperidine-2,6-dione	525893	156
aconitic acid	586815	229
cholesterone	1095940	456
citric acid	617342	273
cytidine-5-monophosphate	700635	243
glucose-1-phosphate	594647	217
glucuronic acid	665901	333
hexadecylglycerol	867593	205
ile-ile	677904	158
inosine	897434	230
inositol-4-monophosphate	845976	315
isopropylbenzene	240619	105
lanosterol	1129573	393
octadecylglycerol	928878	205
shikimic acid	608627	204
sorbitol	667922	217
tocopherol alpha-	1067809	237
UDP GlcNAc	627437	226
UDP-glucuronic acid	587601	217

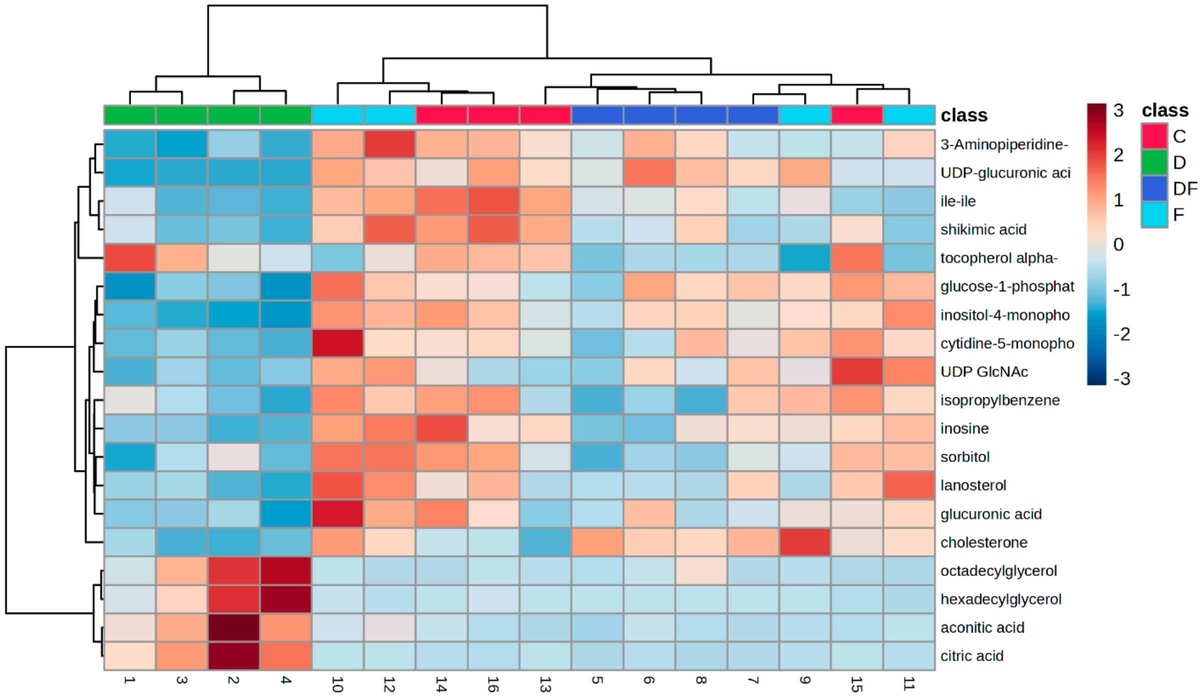

Figure S1. Heatmap of 19 identified metabolites significantly affected by treatment. Each column represents one biological replicate.
